# Molecular mechanisms and clinical significance of the glioma metabolic-immune axis: a comprehensive review

**DOI:** 10.3389/fimmu.2026.1818249

**Published:** 2026-05-19

**Authors:** Jianhuang Huang, Jiexin Huang, Yijing Lin, Caihou Lin

**Affiliations:** 1 Department of Neurosurgery, Affiliated Hospital of Putian University, Putian, Fujian, China; 2 Department of Neurosurgery, Fujian Medical University Union Hospital, Fuzhou, Fujian, China

**Keywords:** glioma, immune evasion, immunotherapy, metabolic reprogramming, metabolic-immune axis, multi-omics, prognostic biomarkers, tumor immune microenvironment

## Abstract

Glioma is the most common and highly aggressive tumor of the central nervous system. Increasing evidence indicates that metabolic reprogramming and the tumor immune microenvironment are intricately interconnected and jointly contribute to tumor initiation, progression, and therapeutic resistance. In recent years, advances in multi-omics technologies have revealed that multiple metabolic pathways—including nucleotide, amino acid, and lipid metabolism—are closely associated with immune cell infiltration, immune evasion, and clinical outcomes in glioma, leading to the emergence of the “metabolic-immune axis” concept. This review systematically summarizes the regulatory mechanisms by which metabolic reprogramming shapes the glioma immune microenvironment and highlights key metabolic genes and immune phenotypes as potential molecular biomarkers for prognosis prediction and immunotherapeutic response. We further discuss multi-omics-based glioma classification strategies, the mechanistic roles of metabolic pathways in immune escape, and the therapeutic potential of combining metabolic-targeted interventions with immunotherapy. By integrating current research advances and existing challenges, this review aims to provide a comprehensive framework for understanding the metabolic-immune interplay in glioma and to identify promising targets for precision therapy and future clinical translation.

## 1 Introduction

Glioma is the most common and highly malignant tumor of the central nervous system, and its marked heterogeneity and aggressive progression continue to pose major challenges for clinical management. Metabolic reprogramming is recognized as a key driver of glioma malignancy, as tumor cells adjust energy metabolism pathways to support rapid proliferation while simultaneously altering metabolic outputs that reshape the tumor immune microenvironment and promote immune evasion (1, 2). Within the tumor microenvironment (TME) of glioma, tumor cells interact with immune cells, vascular cells, and stromal components to form a complex signaling network. This network not only supports tumor growth and invasion but also influences immune cell functional states, leading to the establishment of an immunosuppressive microenvironment (3).

Metabolic pathway remodeling is particularly prominent in glioma. For example, tumor cells enhance glycolytic activity and undergo a metabolic shift from oxidative phosphorylation to aerobic glycolysis, thereby meeting their elevated demands for energy production and biosynthesis (4). In addition, dysregulation of specific metabolic pathways, such as methionine (Met) metabolism, not only promotes tumor cell proliferation but also regulates the polarization of tumor-associated macrophages, leading to the formation of an immunosuppressive microenvironment and further facilitating immune escape (3). The interplay within this metabolic-immune axis provides a new perspective for understanding glioma progression and highlights potential opportunities for targeted therapeutic strategies.

An immunosuppressive tumor microenvironment represents one of the major causes of therapeutic failure in glioma. Tumor cells accumulate metabolic byproducts such as lactate, which reduce extracellular pH, suppress the activity of effector T cells and natural killer (NK) cells, and promote the polarization of tumor-associated macrophages (TAMs) toward the immunosuppressive M2 phenotype, thereby enhancing immune evasion (5, 6). Importantly, beyond its classical role as an acidifying waste product, lactate has recently been recognized as a key signaling molecule through protein lactylation—a novel post-translational modification in which lactyl groups are covalently attached to lysine residues on histones and other proteins. This modification, exemplified by histone H3K18 lactylation (H3K18la), drives epigenetic reprogramming that activates immunosuppressive transcriptional programs in tumor-associated macrophages and modulates T cell exhaustion within the glioma microenvironment (5–7). Lactylation thus represents a direct biochemical link between the Warburg effect and epigenetic immune regulation, substantially expanding our understanding of how metabolic reprogramming sustains the immunosuppressive milieu in glioma. Moreover, metabolic alterations also influence the expression of immune checkpoint molecules, including the upregulation of PD-L1, further impairing the cytotoxic function of immune cells (6). Therefore, a comprehensive understanding of the molecular mechanisms underlying the metabolic-immune axis-including these emerging lactylation-dependent pathways-is essential for overcoming immune escape and therapeutic resistance in glioma.

In recent years, the integration of multi-omics technologies with machine learning approaches has markedly advanced the classification of glioma subtypes associated with metabolic and immune characteristics. By combining transcriptomic data, single-cell RNA sequencing, and spatial transcriptomics, researchers have established classification frameworks based on metabolic and immune features, enabling the identification of distinct molecular subtypes closely associated with clinical outcomes (8, 9). Machine learning algorithms have further improved subtype discrimination and facilitated the identification of key metabolic and immune regulatory genes, providing critical support for prognostic evaluation and the development of personalized therapeutic strategies (9, 10).

Taken together, the pronounced heterogeneity of glioma is closely linked to metabolic reprogramming. Metabolic remodeling not only satisfies the energetic and biosynthetic demands of tumor cells but also promotes immune escape and therapeutic resistance through modulation of the tumor immune microenvironment. Investigation of the metabolic-immune axis offers new insights into the biological characteristics of glioma, while the integration of multi-omics and machine learning approaches continues to drive advances in molecular subtyping and precision therapy. This review systematically summarizes the molecular mechanisms of the metabolic-immune axis in glioma and discusses its clinical significance, with the aim of providing a theoretical basis and practical guidance for future therapeutic strategies.

## 2 Major characteristics of metabolic reprogramming in glioma and their immunoregulatory roles

### 2.1 Glycolysis and lactate metabolism in glioma

Glucose metabolism reprogramming, particularly the shift toward aerobic glycolysis known as the Warburg effect, is one of the most fundamental metabolic characteristics of glioma and plays a central role in shaping the tumor immune microenvironment. Even in the presence of oxygen, glioma cells preferentially convert glucose into lactate rather than fully oxidizing pyruvate through the tricarboxylic acid (TCA) cycle and oxidative phosphorylation. This metabolic adaptation enables rapid ATP generation and provides glycolytic intermediates for biosynthetic pathways required for tumor proliferation (4, 11).

At the molecular level, this glycolytic phenotype is driven by increased expression of glucose transporters and key glycolytic enzymes, including GLUT1 (SLC2A1), HK2, PKM2, and LDHA, under the control of oncogenic signaling pathways such as PI3K/AKT/mTOR, MYC, and HIF-1α (4, 12, 13). In glioma, enhanced glycolysis is closely associated with aggressive biological behavior, treatment resistance, and poor prognosis. Moreover, the high glucose consumption of tumor cells generates a state of metabolic competition within the tumor microenvironment, depriving infiltrating T cells of glucose required for activation, proliferation, and effector function (14).

A major consequence of glycolytic reprogramming is the excessive accumulation of lactate in the tumor microenvironment. Lactate is exported by monocarboxylate transporters, particularly MCT1 (SLC16A1) and MCT4 (SLC16A3), resulting in extracellular acidification (15, 16). The acidic microenvironment directly suppresses the activity of CD8+ T cells and NK cells by impairing cytokine production, T-cell receptor signaling, and cytotoxic granule release, while simultaneously promoting the polarization of tumor-associated macrophages toward the immunosuppressive M2 phenotype (5, 6, 15). Lactate-mediated acidification operates together with nutrient deprivation and immune checkpoint signaling to establish a metabolically hostile milieu for anti-tumor immunity.

Importantly, lactate is now recognized not only as a metabolic byproduct but also as a signaling molecule capable of regulating gene expression through protein lactylation. Histone lactylation provides a direct biochemical link between glycolytic metabolism and epigenetic regulation, promoting transcriptional programs associated with macrophage M2 polarization, immune tolerance, and tumor progression (7, 17, 18). In glioma, emerging evidence suggests that lactylation-related gene signatures are associated with immune infiltration patterns, prognosis, and immunotherapy sensitivity, indicating that the lactate-lactylation axis may serve as both a mechanistic driver and a biomarker of the metabolic-immune state (19, 20).

In addition, glycolysis is tightly linked to immune checkpoint regulation. Lactate accumulation and hypoxia can stabilize HIF-1α, which directly enhances PD-L1 expression on tumor cells and immune suppressor cells, thereby reinforcing T-cell exhaustion and immune evasion (6, 21, 22). These findings indicate that glucose metabolism is not merely a supplier of energy and biomass, but a major regulator of immune escape in glioma.

Taken together, glycolysis and lactate metabolism form a central metabolic axis in glioma, coupling rapid tumor growth with immune suppression through nutrient competition, extracellular acidification, epigenetic remodeling, and checkpoint regulation. Therefore, targeting glycolytic enzymes, lactate transporters, or lactylation-associated pathways may provide promising strategies for combination therapy with immunotherapy in glioma (7, 15, 20).

### 2.2 Roles of pyrimidine and purine metabolism in glioma

Pyrimidine and purine metabolism constitute two core pathways of nucleotide biosynthesis and play critical roles in glioma initiation, progression, and regulation of the immune microenvironment. In recent years, studies integrating gene expression profiling, metabolomics, functional experiments, and clinical data have demonstrated the pivotal contributions of these metabolic pathways to glioma malignancy and patient prognosis.

First, risk models based on pyrimidine metabolism-related genes are closely associated with glioma malignancy and clinical outcomes. A pyrimidine metabolism-related risk score constructed from two human glioma RNA sequencing datasets showed that tumors in the high-risk group exhibited higher malignancy and poorer survival outcomes. This risk score not only serves as an independent prognostic biomarker for glioma but is also closely linked to regulation of the tumor immune microenvironment (23). In addition, stable isotope tracing analyses in glioma tissues and stem-like cells revealed diverse intracellular and extracellular metabolic features of pyrimidine metabolism within the tumor microenvironment, suggesting its involvement in immune regulation and metabolic reprogramming (24, 25). In specific glioma subtypes such as diffuse midline glioma (DMG), a strong dependence on *de novo* pyrimidine biosynthesis has been observed. Accordingly, inhibition of the key enzyme DHODH significantly suppresses tumor growth and prolongs survival in animal models, indicating the therapeutic potential of targeting this pathway (26, 27). At the molecular level, expression of key enzymes in pyrimidine metabolism is associated with tumor cell proliferation and ribosomal RNA synthesis, and inhibition of this pathway induces nucleolar stress and cell-cycle arrest, thereby suppressing glioma cell proliferation (28).

Second, pyrimidine metabolism influences immune cell infiltration and function through modulation of the tumor immune microenvironment. High-risk pyrimidine metabolism gene expression profiles are accompanied by increased immune infiltration, resulting in an immune “hot” tumor phenotype that may provide a biological basis for immune checkpoint inhibitor therapy (23). Moreover, the metabolic states of glioma-associated macrophages and microglia (GAMMs) are closely related to pyrimidine and other nucleotide metabolic activities, and their dynamic changes within the tumor microenvironment influence mechanisms of immune escape (29, 30).

Third, expression of purine metabolism-related genes is positively correlated with immune checkpoint molecules such as PD-1/PD-L1, suggesting that purine metabolism may sustain the immune “hot” state of tumors by regulating checkpoint expression and function. Large multicenter analyses have shown that risk scores derived from purine metabolism-related gene expression are associated not only with glioma invasiveness and prognosis but also with immune infiltration characteristics and immune checkpoint expression, highlighting their potential relevance in tumor immunotherapy (31). In specific mutational contexts, such as H3K27M-mutant diffuse midline glioma, purine metabolism is aberrantly activated, and adaptive reprogramming of this pathway contributes to radiotherapy resistance, whereby inhibition of key enzymes and associated pathways enhances radiosensitivity (32, 33). In addition, metabolites derived from purine metabolism participate in DNA damage repair and cell-cycle regulation, thereby influencing glioma cell proliferation and survival (34).

Collectively, pyrimidine and purine metabolism are not only essential for sustaining rapid proliferation and metabolic adaptation in glioma cells but also contribute to immune escape and modulation of antitumor immune responses by regulating the tumor immune microenvironment, immune cell infiltration, and immune checkpoint expression. Key enzymes within these pathways, such as DHODH and UMPS, represent potential therapeutic targets. In the future, risk models based on pyrimidine and purine metabolism may serve as tools for prognostic evaluation and prediction of immunotherapy responses in glioma, while the development of pharmacological agents targeting these metabolic pathways open new avenues for precision therapy in glioma (31, 35).

### 2.3 Amino acid metabolism and remodeling of the immune microenvironment

Amino acid metabolism plays a crucial role in glioma progression and in shaping the tumor immune microenvironment. Through metabolic reprogramming of amino acid pathways, glioma cells not only meet the energetic and biosynthetic demands of rapid proliferation but also modulate the metabolic states of immune cells, thereby influencing tumor immune escape. Glutamine, one of the most abundant and multifunctional amino acids in the body, serves as an important metabolic substrate for both tumor cells and immune cells within the tumor microenvironment. Glutamine metabolism regulates tumor cell energy supply and biosynthesis while also affecting immune cells—particularly tumor-associated macrophages (TAMs), regulatory T cells (Tregs), and myeloid-derived suppressor cells (MDSCs)—promoting the formation of an immunosuppressive microenvironment and enhancing immune evasion. Consensus clustering analyses based on glutamine metabolism-related gene expression profiles have stratified glioma patients into distinct subtypes with significant differences in immune cell infiltration, immune phenotypes, and prognosis, suggesting that glutamine metabolic reprogramming is closely associated with both malignant progression and responses to immunotherapy (36, 37).

Tyrosine metabolism also plays an important role in immune regulation in glioma. High expression of tyrosine metabolism enzymes has been associated with upregulation of programmed death-ligand 1 (PD-L1), an immune checkpoint molecule that suppresses effector T-cell activity and promotes tumor immune escape. Mechanistically, this regulation occurs through at least two well-characterized pathways. First, tyrosine-derived metabolic intermediates can stabilize HIF-1α by competitively inhibiting prolyl hydroxylase domain (PHD) enzymes, even under normoxic conditions; stabilized HIF-1α subsequently binds the hypoxia-response element (HRE) within the CD274 (PD-L1) promoter, driving its transcriptional upregulation (38–40). Second, tyrosine catabolism generates α-ketoglutarate (α-KG), which participates in JAK/STAT3 signaling cross-talk; phosphorylated STAT3 directly binds regulatory elements in the PD-L1 promoter, further enhancing its expression and establishing a profoundly immunosuppressive milieu (38–40). These mechanisms collectively promote malignant progression of glioma cells and reduce the cytotoxic effects of tumor-infiltrating immune cells. Clinical data further indicate that high expression of tyrosine metabolism-related genes is closely associated with poor prognosis and reduced responsiveness to immunotherapy in patients with glioma (38, 39).

In addition, several studies have constructed risk-scoring models based on amino acid metabolism-related genes, which effectively predict patient survival and responses to immunotherapy. Using transcriptomic data and multi-omics approaches, investigators have identified a series of gene signatures associated with amino acid metabolism and immunity; these signatures reflect both the metabolic state of tumor cells and the characteristics of the tumor immune microenvironment. High risk scores are typically associated with immunosuppressive microenvironments, including enrichment of M2 macrophages and elevated expression of immune checkpoint molecules, and demonstrate low sensitivity to immune checkpoint inhibitor therapy, indicating their potential as predictive markers and evaluation tools for immunotherapeutic efficacy (37, 41).

In summary, amino acid metabolism-particularly glutamine and tyrosine metabolism-occupies a central position in glioma cell energy metabolism and immune cell functional regulation. Through multiple molecular mechanisms, these pathways remodel the tumor immune microenvironment and promote immune escape and tumor progression. Future studies should further elucidate the specific roles of amino acid metabolism-related enzymes and signaling pathways and explore targeted therapeutic strategies against these metabolic routes to improve immunotherapeutic outcomes and clinical prognosis in patients with glioma (36–38).

### 2.4 The dual role of lipid metabolism in immunoregulation of glioma

Lipid metabolism exhibits genuinely complex and bidirectional roles in immunoregulation in glioma, functioning as both a driver of immunosuppression and, under specific conditions, a promoter of anti-tumor immune responses. Understanding this duality is essential for rational exploitation of lipid metabolic pathways as therapeutic targets.

Immunosuppressive roles of lipid metabolism. From the perspective of lipid metabolism-related genes, risk models constructed using multi-omics data indicate that a high lipid metabolic state is closely associated with an immunosuppressive tumor microenvironment. Analyses based on the TCGA and CGGA databases have shown that expression patterns of lipid metabolism-related genes effectively stratify patient survival outcomes, with the high-risk group displaying pronounced immunosuppressive features, including altered immune cell infiltration and upregulation of immune checkpoint genes (42, 43). Fatty acid oxidation (FAO) plays a particularly critical role in tumor-associated macrophages (TAMs). Glioma promotes lipid metabolic activity, especially FAO, thereby inducing TAM polarization toward the M2 phenotype, which exhibits immunosuppressive functions that support tumor growth and immune escape. For example, the pseudogene TMEM198B regulates lipid metabolism-related pathways, including activation of fatty acid synthesis and elongation enzymes (ACLY and ELOVL6), enabling tumor-derived exosomes to promote lipid accumulation and enhanced FAO in macrophages, ultimately inducing M2 polarization and facilitating immune evasion (44). In addition, the ALOX5-5-HETE axis has been shown to promote M2 polarization of glioma-associated microglia/macrophages (GAMs) and increase PD-L1 expression, thereby establishing an immunosuppressive microenvironment (45).

Immune-supportive roles of lipid metabolism. Conversely, accumulating evidence demonstrates that specific lipid metabolites and metabolic programs can actively support anti-tumor immune responses, revealing the other face of this “double-edged sword.” First, fatty acid oxidation (FAO) is the predominant metabolic program sustaining long-lived memory CD8+ T cells and central memory T (Tcm) cells. Experimental evidence demonstrates that promoting FAO in tumor-infiltrating lymphocytes enhances their mitochondrial spare respiratory capacity, persistence, and memory formation within nutrient-depleted tumor microenvironments-effects that are potentially exploitable to improve durable immunotherapy responses (46). Second, certain polyunsaturated fatty acids (PUFAs), including omega-3-derived eicosapentaenoic acid (EPA) and docosahexaenoic acid (DHA) and their specialized pro-resolving mediators (SPMs, such as resolvins and protectins), can enhance effector T cell and NK cell cytotoxicity and promote dendritic cell maturation, thereby reinforcing anti-tumor immune surveillance (47). Third, specific lipid metabolites such as prostaglandin E2 (PGE2) exhibit context-dependent immune effects: while high concentrations of PGE2 generated by overexpressed COX-2 in glioma cells typically suppress T cell responses, physiological concentrations of PGE2 signaling through EP2/EP4 receptors can promote Th1 differentiation and CTL priming under certain conditions (48). Fourth, lipid-derived antigens presented by CD1d molecules to NKT cells can trigger potent innate-adaptive anti-tumor immune bridges, representing another immune-activating dimension of lipid metabolism (49). These immune-supportive functions of lipid metabolism highlight opportunities for therapeutic intervention.

Lipid metabolic targets in combined immunotherapy. Studies of lipid metabolism-related genes such as FABP7 and FABP5 have shown that their upregulation in tumor-associated macrophages regulates lipid metabolism and antigen presentation, thereby influencing immune cell infiltration and patient prognosis (43, 50, 51). Nanobodies targeting ALOX5 have demonstrated the potential to inhibit M2 polarization, improve the immune microenvironment, and enhance the efficacy of PD-1 immune checkpoint inhibitors (45). Furthermore, regulation of lipid metabolic enzymes such as LCAT is closely associated with tumor immune escape and represents a potential therapeutic target (52). Therefore, strategies combining lipid metabolism modulation with immunotherapy provide new avenues for integrated treatment in glioma and may improve therapeutic responses and survival outcomes.

In summary, lipid metabolism plays a genuine “double-edged sword” role in glioma immunoregulation. On one hand, dysregulated lipid metabolism—particularly enhanced FAO in TAMs and arachidonic acid-derived eicosanoids—promotes immunosuppressive M2 polarization, PD-L1 upregulation, and immune evasion. On the other hand, specific lipid species (omega-3 PUFAs, FAO-dependent metabolic programming in memory T cells, and lipid antigen presentation via CD1d) actively support anti-tumor immunity. Precision therapeutic strategies must therefore distinguish between these opposing metabolic contexts, selectively suppressing immunosuppressive lipid pathways while preserving or enhancing immune-supportive lipid functions. Further elucidation of the molecular mechanisms linking specific lipid species and metabolic enzymes to immune regulatory outcomes will aid in the development of precise metabolism-immunity combinatorial strategies and improve clinical management of glioma (42–45).

## 3 Multi-omics integration for metabolic-immune subtyping and clinical implications

### 3.1 Molecular subtyping of gliomas based on metabolic and immune genes

Glioma, a highly heterogeneous brain tumor, exhibits complex interactions between metabolic reprogramming and the immune microenvironment that profoundly influence tumor progression and patient prognosis. In recent years, advances in multi-omics data accumulation and analytical methodologies have established molecular subtyping based on metabolic and immune gene signatures as a pivotal approach for elucidating glioma biology and informing clinical management. By integrating transcriptomic, proteomic, and radiomic data, researchers have identified multiple glioma subtypes associated with metabolic-immune features. These subtypes display marked differences not only in molecular characteristics but also in immune infiltration patterns and clinical outcomes.

First, multi-omics data fusion analyses have revealed four principal metabolic-immune subtypes with distinct metabolic activity and immune response levels. For instance, one study employed non-negative matrix factorization (NMF) and weighted gene co-expression network analysis (WGCNA), leveraging the TCGA, CGGA, and GEO databases, to construct a metabolic gene-based subtyping framework. This approach uncovered significant inter-subtype differences in metabolic pathway activity, immune cell infiltration, and TME status (9). Moreover, subtypes with high metabolic and high immune gene expression typically exhibit an immunosuppressive TME and aggressive tumor progression with poor prognosis, whereas those with low metabolic and low immune expression demonstrate favorable clinical outcomes (53, 54).

Specifically, the metabolic-high/immune-high subtype is frequently accompanied by enrichment of immunosuppressive cells in the TME, such as M2-polarized macrophages and regulatory T cells (Tregs). These cells secrete immunosuppressive factors that promote immune evasion, resulting in markedly reduced patient survival (19, 55). In contrast, the metabolic-low/immune-low subtype manifests as an immunologically “cold” state, characterized by attenuated tumor cell metabolic activity, limited immune cell infiltration, and relatively better prognosis (56, 57). These observations underscore the synergistic role of metabolic and immune states in glioma biology.

Furthermore, machine learning techniques, particularly MRI-based radiomics, offer a non-invasive strategy for predicting metabolic-immune subtypes in gliomas. By extracting textural, morphological, and metabolism-associated imaging features and applying algorithms such as LASSO regression, random forest, and gradient boosting machines, research teams have developed accurate predictive models that bridge imaging phenotypes to molecular subtypes (9, 53). This approach circumvents the invasiveness of traditional tissue biopsy, enables real-time monitoring of tumor molecular dynamics, and supports personalized treatment decisions.

Overall, molecular subtyping of gliomas based on metabolic and immune genes integrates multi-layered omics data to illuminate the intricate interplay between tumor metabolism and the immune microenvironment. Patients with the metabolic-high/immune-high subtype generally have the poorest prognosis, suggesting the need for aggressive immune-modulatory and metabolic-targeted interventions; conversely, those with the metabolic-low/immune-low subtype exhibit better outcomes and may benefit from conservative management and surveillance. Meanwhile, non-invasive subtype prediction via machine learning models provides robust technical support for precise clinical stratification and individualized therapy. These findings deepen our understanding of the glioma metabolic-immune axis and lay a foundation for future development of combined strategies targeting metabolic and immune pathways (9, 53–55).

### 3.2 Proteomics reveals metabolism- and immune-driven subtypes

Proteomic profiling has offered a transformative perspective on glioma molecular classification, with notable advances in delineating metabolism and immunity as two primary driving forces. In a comprehensive analysis of 343 glioma specimens and 53 normal-appearing brain tissues, researchers identified two clearly distinguishable proteomic subtypes: a metabolic-neuronal subtype and an immune-inflammatory subtype. The metabolic-neuronal subtype is characterized by enrichment of multiple metabolic enzymes and neurotransmitter receptor proteins, indicative of elevated metabolic activity and neuron-like phenotypic features. In contrast, the immune-inflammatory subtype exhibits upregulation of immune-related proteins and inflammatory mediators, reflecting prominent immune cell infiltration and inflammatory responses within the tumor microenvironment (TME). These two proteomic subtypes display substantial differences in clinical prognosis: patients with the metabolic-neuronal subtype generally experience more favorable outcomes, whereas those with the immune-inflammatory subtype are associated with significantly poorer survival. Moreover, the subtypes differ markedly in underlying tumorigenesis mechanisms, microenvironmental regulation, and potential therapeutic targets, underscoring the pivotal roles of metabolic and immune processes in glioma biology and patient survival (58).

The seemingly paradoxical observation that the immune-inflammatory subtype—characterized by prominent immune cell infiltration—is associated with significantly poorer prognosis rather than improved anti-tumor immunity warrants mechanistic clarification. This finding is consistent with emerging evidence demonstrating that the functional composition, rather than the mere quantity, of immune infiltrates determines clinical outcomes in glioma. In the immune-inflammatory subtype, the immune infiltrate is dominated not by cytotoxic CD8+ T cells but by immunosuppressive populations, including M2-polarized tumor-associated macrophages (TAMs), regulatory T cells (Tregs), and myeloid-derived suppressor cells (MDSCs), which collectively secrete pro-tumorigenic cytokines (TGF-β, IL-10) while actively suppressing effector immune function (58, 59). Furthermore, the chronic antigen stimulation and metabolic deprivation characteristic of this metabolic-immune milieu drive progressive T cell exhaustion, marked by upregulation of multiple co-inhibitory receptors (PD-1, TIM-3, LAG-3), rendering physically infiltrating T cells functionally impotent (58). Compounding this, persistent inflammatory signaling through IL-6/STAT3 and NF-κB pathways paradoxically promotes tumor cell proliferation, angiogenesis, and invasion—a well-established phenomenon of inflammation-driven tumor promotion. Additionally, the high inflammatory activity in this subtype drives robust upregulation of PD-L1 on tumor cells, further neutralizing any cytotoxic immune response (59, 60). Thus, high immune infiltration in the immune-inflammatory subtype reflects a dysfunctional, immunosuppression-dominated state rather than productive anti-tumor immunity, explaining its association with inferior survival outcomes compared to the metabolic-neuronal subtype.

With respect to metabolic regulation, proteomic studies have uncovered aberrant expression of key enzymes, particularly dihydropyrimidine dehydrogenase (DPYD) and thymidine phosphorylase (TYMP), both of which are tightly linked to nucleotide metabolism reprogramming. These enzymes are significantly upregulated in the metabolic-neuronal subtype, highlighting the reliance of tumor cells on nucleotide pathway reprogramming to fulfill the biosynthetic and proliferative demands of rapid growth. Functional validation experiments have demonstrated that DPYD and TYMP not only serve as promising prognostic biomarkers but also represent actionable therapeutic targets; pharmacological inhibition of nucleotide metabolism effectively restrains tumor proliferation and recurrence. These findings provide a robust theoretical and experimental foundation for the development of novel anti-glioma agents directed against metabolic pathways (58). Furthermore, proteomic analyses of low-grade gliomas (LGG) corroborate the differential expression of metabolic proteins across distinct molecular subtypes, reinforcing the central importance of metabolic dysregulation in glioma subtyping (61).

Conversely, gliomas of the immune-inflammatory subtype display increased immune cell infiltration, elevated inflammatory cytokine levels, and enhanced expression of immunomodulatory molecules, collectively suggesting an activated immune state in the TME. This subtype may exhibit heightened sensitivity to immunotherapy; however, it simultaneously confronts challenges posed by immune evasion mechanisms. Relevant investigations have revealed that chemokines such as CXCL14 are highly expressed in the immune-inflammatory subtype, where they promote CD8+ T cell chemotaxis and augment anti-tumor immune responses, ultimately contributing to improved patient survival (59). Additionally, the integration of proteomics with spatial omics and other multi-omics modalities has enabled detailed characterization of the complex immune microenvironment in immune-subtype gliomas, elucidating immunosuppressive pathways and identifying candidate targets for immunotherapy (60, 62).

In summary, proteomics has not only delineated two major metabolism- and immunity-driven subtypes in gliomas but has also clearly delineated their pronounced differences in biological characteristics, clinical prognosis, and therapeutic vulnerabilities. Notably, DPYD and TYMP emerge as critical enzymes in nucleotide metabolism reprogramming, opening new avenues for precision-targeted therapy. These insights establish a solid foundation for advancing glioma molecular classification, prognostic stratification, and personalized treatment strategies, while directing future research toward a deeper exploration of the interactive mechanisms within the metabolic-immune axis and their translational potential (60, 61).

Metabolic enzymes and their products play essential roles in regulating immune checkpoint expression. Altered metabolic pathways in glioma can upregulate checkpoint molecules, including PD-L1, through intracellular metabolic intermediates and signaling cascades, thereby suppressing immune cell cytotoxicity. Additionally, metabolites can modulate immune cell differentiation and function through pathways such as mTOR signaling and epigenetic modifications, further contributing to immune tolerance and reduced anti-tumor immunity.

## 4 Mechanisms of metabolic product-mediated immune evasion

### 4.1 Impact of metabolic products on immune cell function

Tumor cells profoundly suppress anti-tumor immunity in the tumor microenvironment (TME) by secreting a diverse array of metabolic byproducts, primarily targeting key effector immune cells such as macrophages, dendritic cells (DCs), and effector T cells. These metabolites not only exert direct inhibitory effects on immune cell function but also promote the expansion of immunosuppressive populations, including regulatory T cells (Tregs) and myeloid-derived suppressor cells (MDSCs), thereby fostering immune tolerance, establishing an immune-evasive TME, and impeding effective anti-tumor immunity.

Lactate and lactylation-mediated immunosuppression. Tumor metabolic reprogramming leads to excessive accumulation of lactate in the TME through enhanced aerobic glycolysis. Lactate reduces local pH and directly impairs immune cells, inhibiting their proliferation and cytokine production, thereby attenuating anti-tumor effector functions (17). Beyond its classical acidifying role, lactate has recently been recognized as a key epigenetic regulator through protein lactylation—a post-translational modification in which lactyl groups are covalently attached to lysine residues on histones (e.g., H3K18la) and non-histone proteins. In the glioma TME, histone lactylation drives transcriptional programs that promote M2-like macrophage polarization, sustain immune-tolerant gene expression, and impair effector T cell function (7, 63). This lactylation axis thus represents a direct biochemical bridge between the Warburg effect and epigenetic immune reprogramming, substantially expanding the mechanistic repertoire through which metabolic reprogramming enforces immunosuppression.

Purine metabolites and the CD39/CD73-adenosine axis. Purine metabolites, particularly extracellular ATP and its catabolite adenosine, constitute a critical and previously underappreciated axis of metabolic immunosuppression in glioma. Under conditions of cellular stress and hypoxia, tumor cells and dying cells release abundant extracellular ATP, which is rapidly catabolized to adenosine by the ectonucleotidases CD39 (NTPDase1) and CD73 (5’-nucleotidase) expressed on tumor cells, TAMs, Tregs, and endothelial cells. The resultant adenosine binds to A2A and A2B adenosine receptors on T cells, NK cells, and dendritic cells, triggering cAMP-mediated signaling that profoundly suppresses immune cell activation, cytokine production (IFN-γ, TNF-α), and cytotoxic effector function (64, 65). Simultaneously, the CD39/CD73 axis promotes Treg expansion and stabilizes their suppressive phenotype. In glioma, CD39 and CD73 are overexpressed on tumor-infiltrating immune cells, and elevated adenosine concentrations in the TME correlate with immunosuppressive microenvironment characteristics and poor prognosis, establishing the CD39/CD73-adenosine pathway as a prime immunotherapeutic target (63).

Tryptophan catabolism and kynurenine-AhR signaling. Tumor cells overexpress indoleamine 2,3-dioxygenase (IDO) and tryptophan 2,3-dioxygenase (TDO), which catalyze tryptophan degradation to produce the immunosuppressive metabolite kynurenine. Kynurenine activates the aryl hydrocarbon receptor (AhR) signaling pathway, suppressing T cell activation while promoting Treg expansion (66, 67). This pathway is particularly relevant in glioma, where IDO/TDO expression is frequently elevated and correlates with immune evasion and treatment resistance (66).

Glutamine deprivation and metabolic competition. Glutamine metabolism regulates immune cell function through multiple mechanisms that extend beyond its biosynthetic roles. First, competitive glutamine deprivation within the TME directly impairs T cell activation and proliferation, as glutamine is an essential substrate for nucleotide synthesis, TCA cycle anaplerosis, and mTORC1-dependent anabolic metabolism required for T cell expansion (68). Second, glutamine-derived α-ketoglutarate (α-KG) serves as a substrate for α-KG-dependent dioxygenases, including TET demethylases and histone demethylases, thereby modulating macrophage polarization through epigenetic mechanisms. Specifically, α-KG supplementation promotes M1-like macrophage polarization, while glutamine deprivation—by reducing α-KG availability—favors the M2 immunosuppressive phenotype (69). Third, glutamine regulates immune-tumor metabolic competition via mTOR signaling: tumor cells with upregulated glutamine transporters (ASCT2/SLC1A5) outcompete infiltrating T cells for glutamine, suppressing mTORC1 activity in T cells and thereby blunting their activation, proliferation, and effector differentiation (70). These multilayered roles of glutamine in metabolic immune competition underscore its significance in the glioma metabolic-immune axis.

Fatty acid derivatives and broader immune dysfunction. Fatty acid metabolism-derived products such as dihydroxyoctadecenoic acid (DiHOME) have been shown to drive excessive neutrophil activation while impairing monocyte and macrophage function, resulting in broad immune cell dysfunction (71). Furthermore, lipid-derived prostaglandins (particularly PGE2) generated through COX-2-mediated arachidonic acid metabolism suppress dendritic cell maturation and T cell priming, further dampening anti-tumor immune responses.

Suppression of dendritic cells and expansion of immunosuppressive populations. Suppression of dendritic cells compromises their antigen-presenting capacity, leading to inadequate activation of effector T cells and further weakening the immune system’s ability to recognize and eliminate tumor cells (72, 73). In effector T cells, metabolites influence metabolic state and function via diverse pathways; for instance, certain metabolites modulate mTOR signaling and histone modifications, altering T cell differentiation and activation, which reduces cytotoxicity and proliferative capacity (17, 74). Furthermore, tumor-derived metabolites induce expansion of immunosuppressive cell populations, particularly Tregs and MDSCs. These cells secrete immunosuppressive cytokines (e.g., IL-10, TGF-β), further dampening effector immune cell function and reinforcing immune tolerance (73, 75).

Gut microbiota-derived metabolites. Growing attention has focused on the immunomodulatory role of gut microbiota and their metabolites. Microbial metabolites—including short-chain fatty acids (SCFAs), bile acid derivatives, and tryptophan catabolites—regulate T cells, B cells, dendritic cells, and macrophages by activating surface receptors and mediating epigenetic modifications, thereby shaping the tumor immune microenvironment (76–78). These gut-derived metabolites can promote immune homeostasis and suppress inflammation or, under certain conditions, enhance immunosuppression, influencing tumor initiation and progression (79, 80).

**Figure 1:**
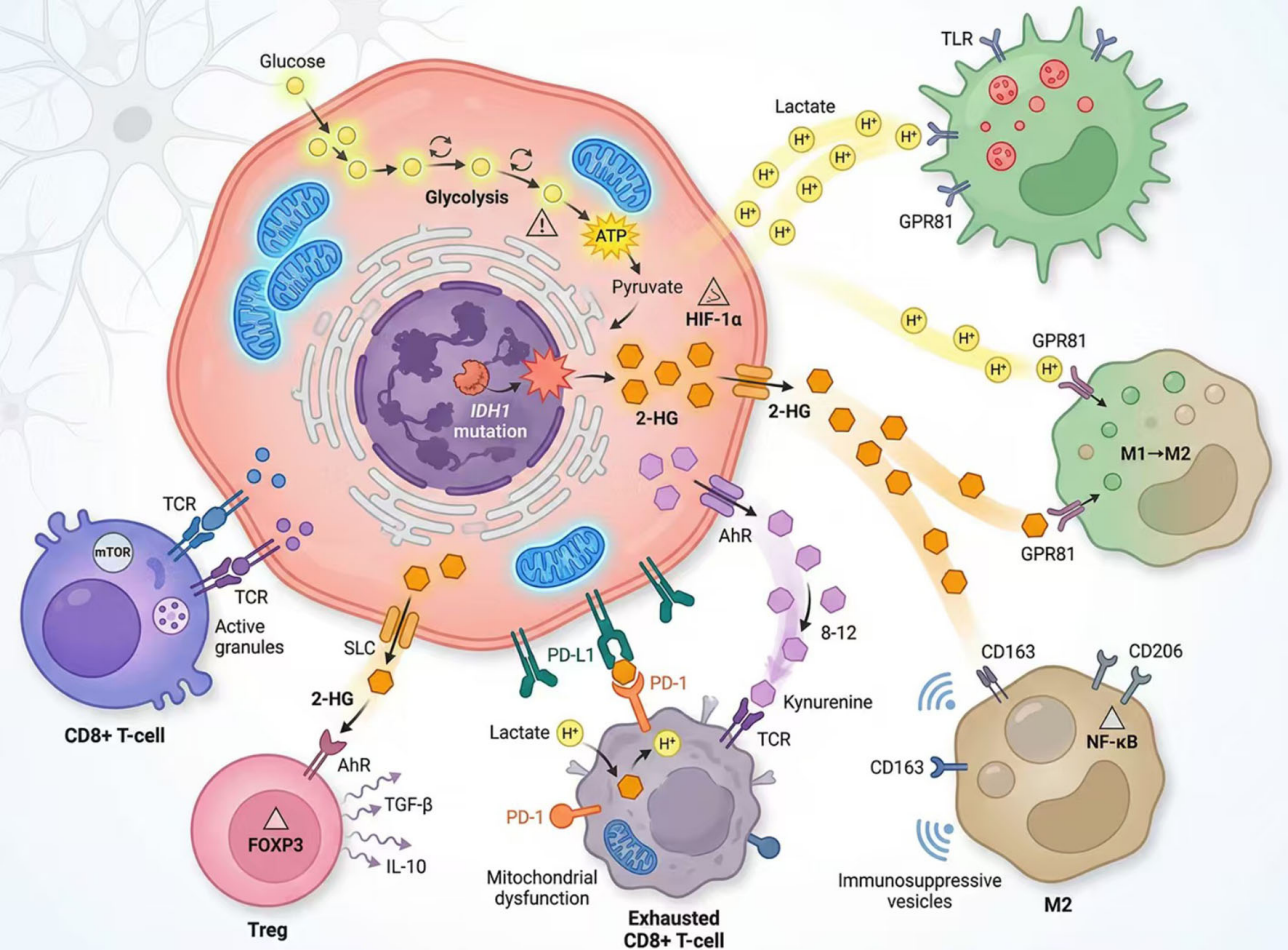
Graphical abstract of the glioma metabolic-immune axis. This schematic summarizes the core concept of how metabolic reprogramming in glioma cells—such as enhanced glycolysis and IDH1 mutation—generates oncometabolites (e.g., lactate, 2-hydroxyglutarate [2-HG], and kynurenine). These metabolites reshape the tumor immune microenvironment by polarizing tumor-associated macrophages toward an immunosuppressive M2 phenotype, inducing T cell exhaustion, and ultimately promoting immune evasion.

In summary, through metabolic reprogramming, tumor cells generate a repertoire of metabolites—including lactate (acting through both pH-dependent and lactylation-dependent mechanisms), purine-derived adenosine (through the CD39/CD73 axis), tryptophan-derived kynurenine (through IDO/TDO-AhR signaling), and glutamine deprivation (through mTOR-dependent metabolic competition)—that collectively inhibit the anti-tumor functions of macrophages, dendritic cells, and effector T cells while driving expansion of regulatory T cells and MDSCs (**Figure 1**). These interactions within the metabolic-immune axis illuminate key mechanisms of tumor immune evasion and highlight promising combinatorial therapeutic strategies targeting metabolic pathways and immune cell function (17, 66, 73, 75).

### 4.2 Metabolic enzymes regulate immune checkpoint expression

Metabolic enzymes play a pivotal role in orchestrating tumor immune evasion, particularly by modulating the expression of immune checkpoint molecules to promote immunosuppression in tumor cells. In gliomas, which exhibit profound metabolic reprogramming, aberrant expression of metabolic enzymes not only sustains tumor cell growth and survival but also enhances immune evasion and diminishes the efficacy of immunotherapy by regulating immune checkpoints such as PD-L1. Tyrosine metabolism and its metabolites exert particularly unique regulatory effects in this process, influencing intracellular key metabolic intermediates that further control immune checkpoint expression and bolster tumor escape from immune surveillance. Concurrently, the oncometabolite (R)-2-hydroxyglutarate (D-2HG), produced as a result of isocitrate dehydrogenase 1 (IDH1) mutations, profoundly alters the tumor immune microenvironment and suppresses immune cell activity, offering novel insights and therapeutic targets for immunometabolic regulation in glioma.

Tyrosine metabolism features distinctive mechanisms in tumor immune modulation. Specifically, α-ketoglutarate (α-KG), a critical intermediate in the tricarboxylic acid (TCA) cycle generated during tyrosine catabolism, participates not only in energy metabolism but also serves as an essential cofactor for epigenetic enzymes, thereby influencing the expression of tumor immune checkpoint molecules. Active tyrosine metabolism drives fluctuations in α-KG levels, which in turn modulate epigenetic enzyme activity and enhance the transcriptional activity of PD-L1, leading to its upregulation and augmented tumor immune evasion. For example, the NAD+ metabolism enzyme nicotinamide phosphoribosyltransferase (NAMPT) sustains α-KG levels, thereby regulating TET1 enzyme activity and promoting IFN-γ-induced PD-L1 expression, ultimately facilitating tumor immune escape (21). Furthermore, studies have demonstrated that manipulating tyrosine metabolism-related pathways can alter immune checkpoint expression levels, highlighting a close link between metabolic enzymes and immune evasion. Dysregulated tyrosine metabolism in glioma cells may promote PD-L1 expression through analogous mechanisms, thereby suppressing effector T cell activity, establishing an immunosuppressive microenvironment, and increasing the tumor’s immune evasion potential. Consequently, targeted modulation of tyrosine metabolism and its products, such as α-KG, represents a promising strategy for reversing immunosuppression and potentiating immunotherapy efficacy.

IDH1 gene mutations represent one of the most common metabolic alterations in glioma, leading to aberrant accumulation of the neomorphic metabolite D-2HG. As an oncometabolite and “metabolic signaling molecule,” D-2HG not only disrupts tumor cell metabolism but also profoundly reshapes the tumor immune microenvironment by modulating immune cell function. Mechanistically, D-2HG inhibits the activity of T cells and dendritic cells, attenuating anti-tumor immune responses and promoting immune evasion. Relevant studies have shown that D-2HG regulates the expression of immunosuppressive cytokines, fosters an immune-tolerant microenvironment, and suppresses CD8+ T cell cytotoxicity and proliferation (81). Moreover, D-2HG may indirectly regulate immune-related gene expression through epigenetic modifications of histones and DNA, reinforcing the expression of immune checkpoint molecules such as PD-L1 and further dampening immune cell activity. Given the dual immunosuppressive functions of D-2HG in IDH1-mutant gliomas, targeted interventions against IDH1 mutations or the D-2HG metabolic pathway have emerged as a major focus in current glioma immunotherapy research. These approaches aim to alleviate D-2HG-mediated immunosuppression and enhance the therapeutic efficacy of immune checkpoint inhibitors.

**Figure 2:**
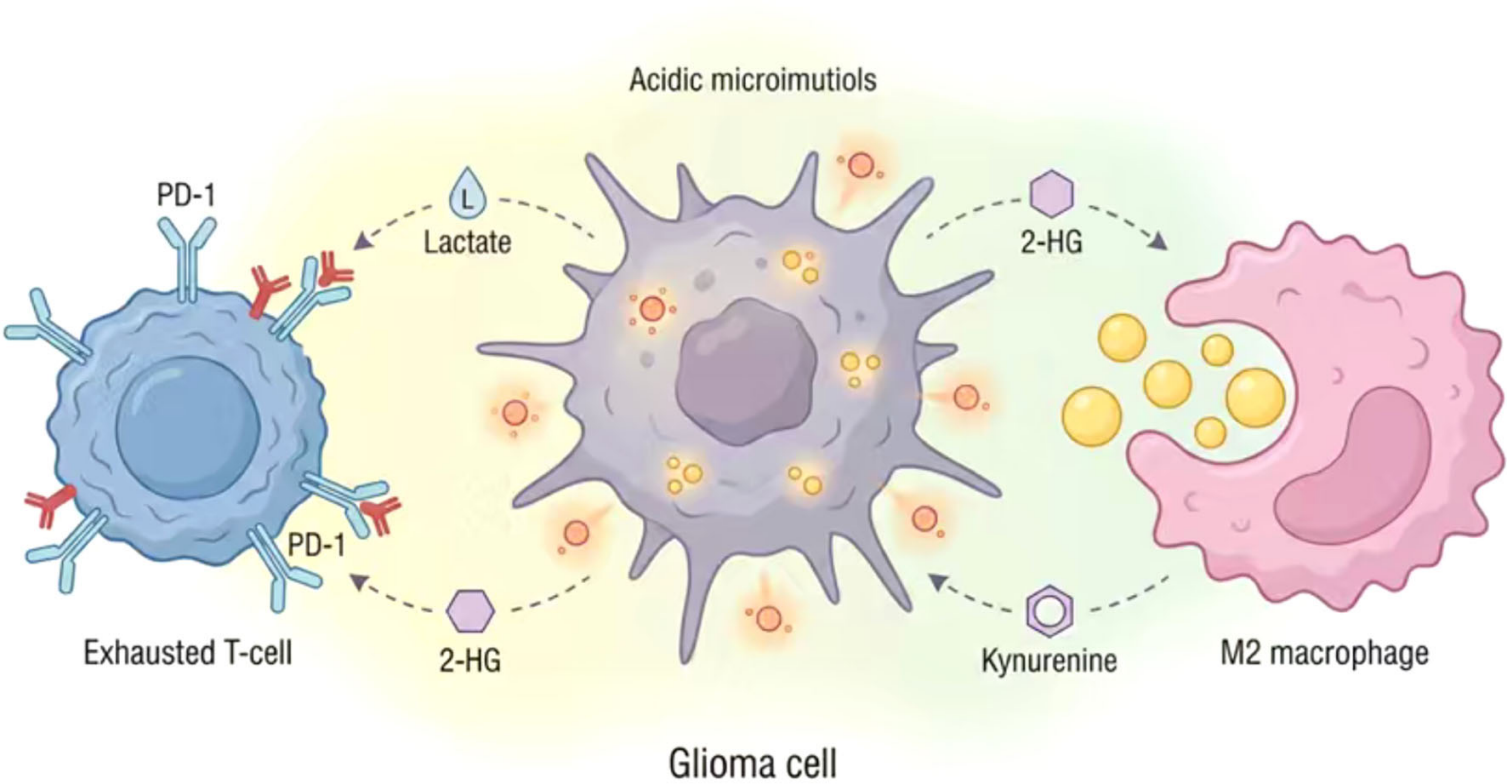
Metabolic-immune interaction landscape driving immune evasion in glioma. This schematic illustrates the complex metabolic crosstalk between glioma cells and the immune microenvironment that collectively fosters an immunosuppressive state. Glioma cells exhibit enhanced aerobic glycolysis (Warburg effect) and frequently harbor IDH1 mutations, resulting in excessive extracellular accumulation of tumor-derived metabolites such as lactate and (R)-2-hydroxyglutarate (2-HG). These metabolites, along with aberrantly synthesized fatty acids, are taken up by tumor-associated macrophages (TAMs), promoting their polarization toward a pro-tumorigenic M2 phenotype. This polarization is typically mediated by signaling pathways involving HIF-1α and PPAR-γ. The lactate-driven acidification of the microenvironment (low pH) synergizes with tryptophan depletion and kynurenine accumulation (mediated by IDO/TDO enzymes) to directly suppress effector T cell proliferation and cytotoxic function. This metabolic stress further upregulates PD-1 expression on T cell surfaces, facilitating immune checkpoint evasion. In turn, immunosuppressive cells secrete cytokines (e.g., TGF-β, IL-10) that reinforce glioma cell survival and metabolic plasticity, perpetuating a vicious cycle that drives tumor progression.

In summary, IDH1 mutations and their metabolite D-2HG exert multi-layered control over the tumor immune microenvironment, serving as a critical regulatory node in the glioma metabolic-immune axis. The above findings collectively indicate that glioma-derived metabolites do not function independently; rather, they form an interconnected metabolic-immune network that simultaneously reprograms tumor cells and immune populations. This integrated interaction landscape is summarized in **Figure 2**.

## 5 Association of metabolic pathways with immunotherapy response

### 5.1 Metabolic risk models for predicting immunotherapy efficacy

**Figure 3:**
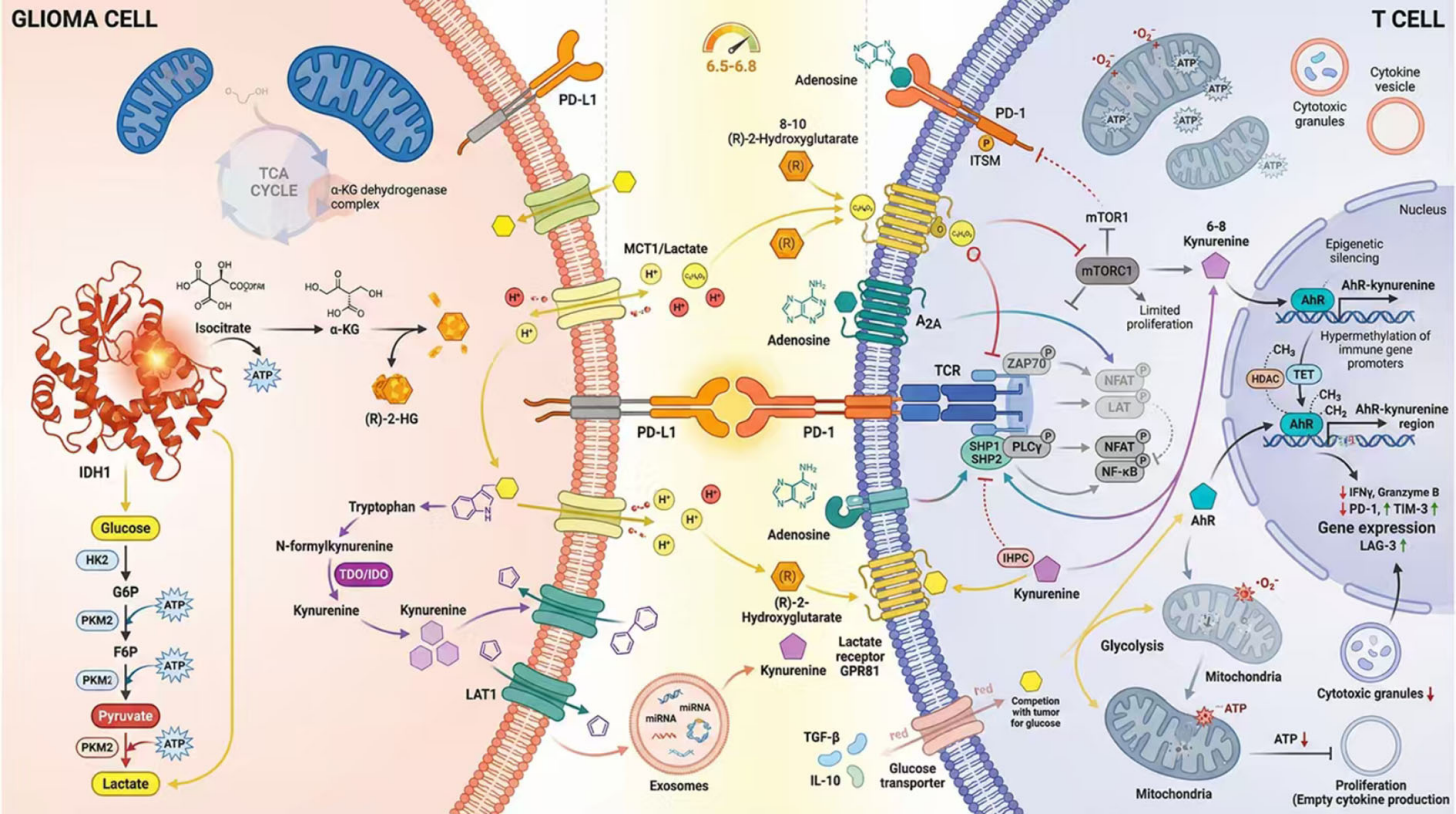
Molecular mechanisms of metabolite-mediated immune evasion at the immunological synapse. The glioma cell overexpresses glucose transporters (GLUT) and glycolytic enzymes to sustain high energy demands, releasing lactate via MCT1/4 transporters. The IDH1 mutant enzyme catalyzes the conversion of α-KG to the oncometabolite (R)-2-HG. Concurrently, tryptophan is catabolized into kynurenine by IDO/TDO enzymes. The synaptic cleft becomes acidic (low pH) due to lactate accumulation. Metabolic impact on T cells (1): Lactate enters T cells via MCT1, causing intracellular acidification and inhibiting the mTOR signaling pathway, which blunts T cell activation and proliferation. (2) (R)-2-HG uptake disrupts mitochondrial respiration and acts as an epigenetic modifier, leading to hypermethylation of effector gene promoters. (3) Kynurenine binds to the Aryl Hydrocarbon Receptor (AhR), which translocates to the nucleus to transcriptionally upregulate PD-1 expression. (4) Checkpoint Engagement: The resulting upregulation of PD-1 binds to PD-L1 on the glioma surface, delivering a potent inhibitory signal (indicated by red inhibitory arrows) that synergizes with metabolic stress to enforce T cell exhaustion and immune evasion.

In recent years, the interplay between tumor metabolic reprogramming and the immune microenvironment has emerged as a central focus in cancer therapy research. Risk scoring models constructed from metabolism-related genes have demonstrated robust capacity to predict patient responses to immune checkpoint inhibitors (ICIs) and other forms of immunotherapy, thereby providing a theoretical foundation for precision oncology. To further illustrate how these metabolites converge on T-cell dysfunction at the immunological synapse, **Figure 3** summarizes the major molecular events, including lactate-mediated intracellular acidification and mTOR suppression, kynurenine-AhR-PD-1 signaling, and (R)-2-HG-associated mitochondrial and epigenetic impairment.

First, lipid metabolism-related gene signatures have exhibited excellent prognostic performance across multiple tumor types, including hepatocellular carcinoma (HCC). A model centered on six lipid metabolism genes (ADH4, LCAT, CYP2C9, CYP17A1, LPCAT1, and ACACA) not only effectively stratifies patients according to survival risk but also reveals that the high-risk group is characterized by an immunosuppressive microenvironment, manifested as immune cell dysfunction and elevated expression of immune checkpoint molecules. Intriguingly, despite these immunosuppressive features, high-risk patients often exhibit superior responses to immune checkpoint blockade, suggesting that lipid metabolism dysregulation may sensitize tumors to immunotherapy (82). Similarly, a risk model integrating fatty acid metabolism and ferroptosis-related genes in prostate cancer successfully predicted differential immunotherapy responses, with the high-risk group displaying higher tumor mutation burden (TMB) and immune evasion characteristics (83).

Risk models based on amino acid metabolism likewise hold substantial clinical relevance. For instance, in pheochromocytoma/paraganglioma (PPGL), a model constructed from six key amino acid metabolism genes (DDC, SYT11, GCLM, PSMB7, TYRO3, and AGMAT) accurately predicts immune infiltration levels and immunotherapy benefit. Although the high-risk group shows relatively lower immune infiltration, it paradoxically demonstrates potentially better immunotherapy responses (84). In glioma specifically, a tryptophan metabolism gene-based risk score is closely correlated with immune infiltration and ICI response; high-risk patients exhibit elevated TMB and increased immune cell infiltration, underscoring the intricate crosstalk between metabolic pathways and the immune microenvironment (85).

Furthermore, lipid metabolism-related long non-coding RNA (lncRNA) risk models have been validated as independent prognostic indicators in gastric cancer, where the high-risk group is associated with upregulated immune checkpoint expression, increased immunosuppressive cell infiltration, and reduced TMB, predicting poorer immunotherapy response (86). In lung adenocarcinoma, a lipid metabolism gene-based model (including PTDSS1) not only predicts survival but also delineates immunosuppressive microenvironment features and differential immunotherapy responses in high-risk patients; functional validation of PTDSS1 further confirmed its role in tumor cell proliferation and apoptosis (87).

In the glutamine metabolism domain, risk models in lung adenocarcinoma have likewise demonstrated predictive value for immunotherapy efficacy. Low-risk patients exhibit an immunologically “hot” phenotype and superior ICI sensitivity, revealing heterogeneity in glutamine metabolism between tumor and immune cells and its impact on immune regulation (88). Similarly, glutamine metabolism-based risk scores in bladder cancer effectively predict prognosis and immunotherapy response, with the key regulatory gene PYCR1 shown to promote tumor progression via metabolic pathway modulation (89).

Risk models derived from pyrimidine and purine metabolism genes have consistently shown tight associations with the immune microenvironment and immunotherapy response in cancers such as HCC, colorectal cancer, and lung cancer. For example, bile acid metabolism-related gene models distinguish prognostic subtypes in liver cancer and predict immunotherapy outcomes (90). Glucose metabolism-related models in colon cancer have revealed immunosuppressive states and immunotherapy resistance in high-risk groups (91, 92). Collectively, these models highlight the interwoven nature of metabolic pathways and immune regulation, positioning metabolic gene signatures as promising biomarkers for immunotherapy response.

High metabolic risk groups are typically characterized by an immunosuppressive TME, featuring immune cell dysfunction, elevated immune checkpoint expression, and tumor immune evasion, which collectively contribute to inferior immunotherapy responses. For instance, high-risk lung adenocarcinoma patients display elevated TIDE scores, indicative of immunotherapy resistance (83, 93). In gastric cancer, high metabolic risk is associated with increased infiltration of immunosuppressive cells and markedly upregulated checkpoint expression, limiting immunotherapy efficacy (86, 94). Nevertheless, combination strategies targeting specific metabolic vulnerabilities can reverse this immunosuppressive state and enhance immunotherapy outcomes. For example, targeting SLC22A5-mediated carnitine metabolism has been shown to overcome immunotherapy resistance in non-small cell lung cancer (95). Similarly, modulation of lipid metabolism genes such as PTDSS1 or glutamine metabolism regulators such as PYCR1 has been validated to suppress tumor growth, reshape the immune microenvironment, and improve immunotherapy efficacy (87, 89). Thus, metabolism-related risk models not only possess predictive power for immunotherapy response but also open new avenues for rational combination of metabolic-targeted and immunotherapeutic strategies.

In summary, risk scoring models constructed from pyrimidine, purine, amino acid, and lipid metabolism genes have been validated across multiple tumor types to correlate strongly with immunotherapy efficacy. High metabolic risk is generally linked to an immunosuppressive microenvironment and poorer immunotherapy response; however, targeted metabolic interventions hold promise for reversing immunosuppression and improving therapeutic outcomes. These findings provide valuable insights for glioma and other tumors within the metabolic-immune axis framework, advancing the precise integration of metabolic targeting with immunotherapy and driving individualized treatment development.

### 5.2 Metabolic targeting combined with immunotherapy strategies

**Figure 4:**
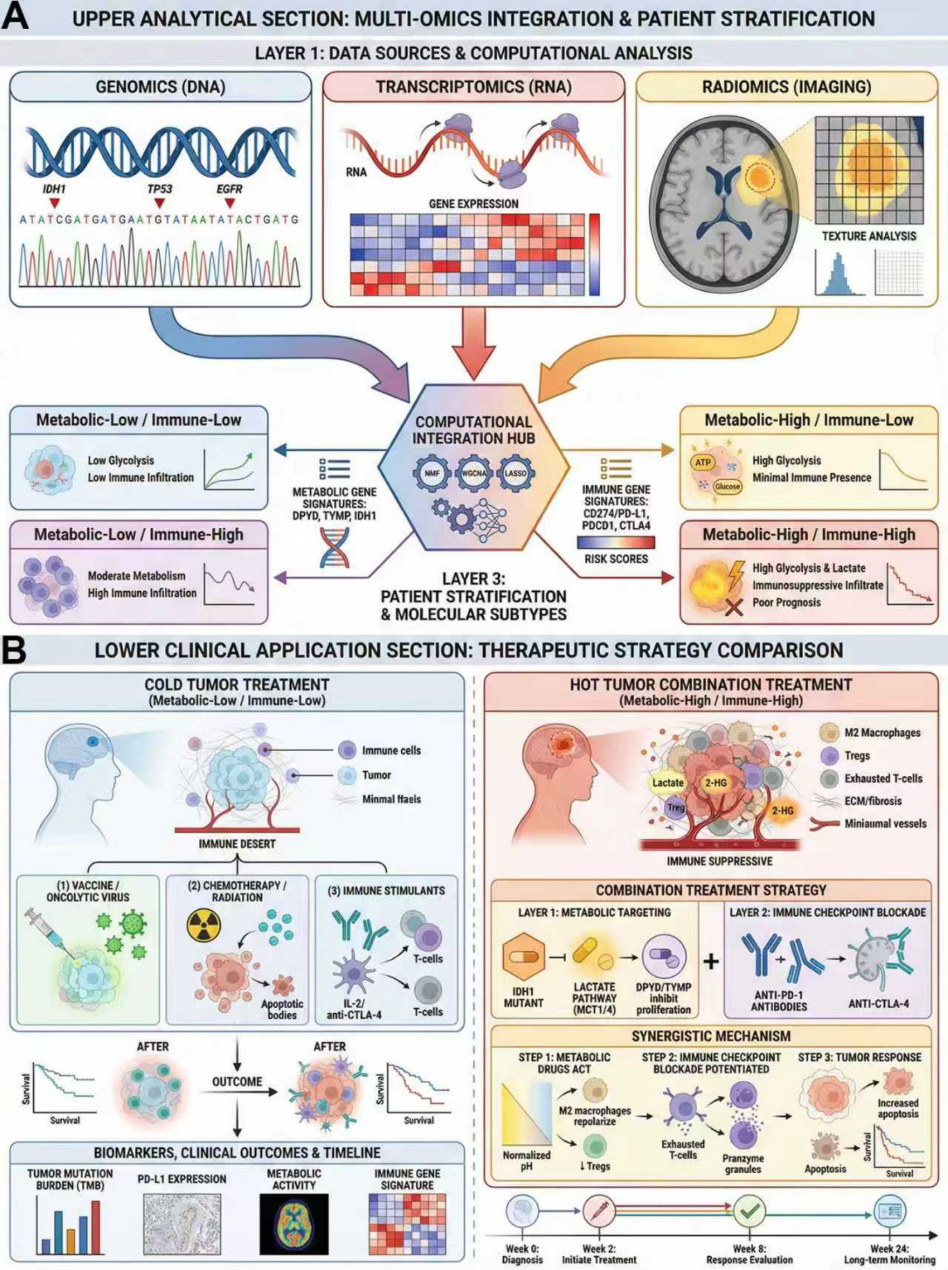
Multi-omics integration for glioma subtyping and precision metabolic-immune combination therapy. **(A)** Multi-Omics Stratification Framework. Integration of high-dimensional data—including genomics (IDH mutation status), transcriptomics (metabolic and immune gene signatures), and radiomics (MRI features)—via machine learning algorithms stratifies glioma patients into four distinct metabolic-immune subtypes. The “Metabolic-High/Immune-High” subtype is identified as having the poorest prognosis due to a metabolically active and highly immunosuppressive microenvironment, representing a prime target for combination therapy. **(B)** Therapeutic Strategy. Schematic comparison of treatment outcomes for the high-risk subtype. The tumor microenvironment remains “cold” or immunosuppressive; T cells are excluded or exhausted due to metabolic barriers. A synergistic approach combining metabolic inhibitors (e.g., IDH1 inhibitors, MCT blockers) with immune checkpoint inhibitors (ICIs, e.g., anti-PD-1 antibodies). Metabolic targeting reduces oncometabolite levels (2-HG, lactate), reversing M2 macrophage polarization and alleviating T cell metabolic suppression. This “metabolic priming” sensitizes the tumor to ICIs, facilitating robust CD8+ T cell infiltration and activation (turning the tumor “hot”), ultimately leading to tumor regression and improved survival outcomes.

Metabolic-targeted therapy combined with immunotherapy represents a frontier direction in current glioma treatment research. This approach aims to modulate metabolic abnormalities in both tumor cells and the immune microenvironment to enhance immunotherapy efficacy and overcome the limitations of conventional monotherapies. Glioma, the most common and prognostically dismal malignancy of the central nervous system, is notoriously refractory to standard treatments due to the blood-brain barrier and its profoundly immunosuppressive tumor microenvironment. Recent studies have demonstrated that multiple metabolic pathways contribute to glioma initiation, progression, and immune evasion, and the combination of metabolic-targeted agents with immunotherapy has shown synergistic potential. Building on these mechanistic insights, **Figure 4** presents a translational framework linking multi-omics-based metabolic-immune subtyping to precision combination strategies integrating metabolic intervention with immunotherapy.

First, metabolic intervention targeting IDH1-mutant gliomas, when combined with radiotherapy and immune checkpoint inhibitors (e.g., anti-PD-L1 antibodies), has achieved significant efficacy in preclinical mouse glioma models. IDH1 mutation is a defining molecular feature of glioma; the mutant enzyme produces (R)-2-hydroxyglutarate (2-HG), which directly reshapes the tumor immune microenvironment, suppresses T cell function, and promotes immune evasion. Research has shown that IDH1 inhibitors combined with radiotherapy and anti-PD-L1 therapy markedly prolong survival in mice, alleviate T cell exhaustion, restore anti-tumor immune activity, and inhibit tumor progression (7, 96). This combinatorial strategy not only targets metabolic enzyme function but also activates the immune system, delivering a dual attack on glioma and addressing the shortcomings of monotherapy.

Second, targeting other metabolic enzymes has also shown promise for synergistic immunotherapy effects. For example, dihydropyrimidine dehydrogenase (DPYD) and thymidine phosphorylase (TYMP), which are aberrantly expressed in glioma and participate in pyrimidine and thymidine metabolism, influence tumor cell proliferation and the immune microenvironment. Studies have demonstrated that inhibition of DPYD and TYMP not only suppresses tumor metabolic activity but also modulates immune cell infiltration and immune checkpoint expression, thereby enhancing immunotherapy efficacy (40, 85). Combining targeted inhibition of these metabolic enzymes with immune checkpoint blockade holds potential to become a novel therapeutic paradigm in glioma.

Furthermore, metabolic byproducts such as lactate and lactate-mediated histone lactylation play critical regulatory roles in glioma immune evasion and therapy resistance. By targeting lactate-metabolizing enzymes and lactate transporters (e.g., SLC16A1 and SLC16A3), it is possible to improve the tumor immune microenvironment, alleviate immunosuppression, and increase immunotherapy response rates (15, 20). The integration of such metabolic-targeted agents with immunotherapy can effectively reverse tumor-induced immunosuppression, promote T cell infiltration and activation, and enhance therapeutic outcomes.

To strengthen the clinical relevance of immunometabolic strategies, we further summarized representative ongoing or completed clinical trials evaluating metabolic-targeted therapy combined with immunotherapy in glioma and related solid tumors (**Supplementary Table 1**). Although the number of glioma-specific trials remains limited, early-phase studies involving IDH inhibitors, IDO pathway inhibitors, and adenosine-axis modulators have begun to test the feasibility of combining metabolic intervention with immune checkpoint blockade. These trials reflect an important translational trend: metabolic therapies are increasingly being explored not only for direct anti-tumor effects but also as immune-conditioning agents capable of reshaping the tumor microenvironment. At present, most available studies are still in early development, and definitive efficacy data remain limited. Nevertheless, these clinical efforts provide an essential bridge between mechanistic discoveries and future precision immunometabolic therapy in glioma.

In summary, metabolic abnormalities in glioma are intimately linked to immune evasion, positioning combined metabolic-targeted and immunotherapy strategies as a major emerging focus in glioma research. Inhibition of key metabolic enzymes (e.g., IDH1, DPYD, TYMP) and modulation of metabolite production and transport (e.g., lactate) can suppress tumor metabolic activity, remodel the immune microenvironment, reduce T cell exhaustion, and potentiate immunotherapy efficacy. This metabolic-immune axis-based combination approach offers new therapeutic options for glioma patients. Future validation through larger-scale clinical trials will be essential to confirm safety and efficacy and facilitate clinical translation.

## 6 Impact of gut microbiota metabolism on immune regulation in glioma

### 6.1 The gut-brain axis and the glioma immune microenvironment

The gut-brain axis represents a bidirectional communication network linking the gut microbiota to the central nervous system (CNS) and has garnered substantial attention in recent years for its roles in diverse neurological disorders and brain tumors. Glioma, the most prevalent and highly lethal malignancy of the CNS, exhibits a strong association between its pathogenesis, progression, and the composition and function of the gut microbiota. The gut microbiota modulates the intratumoral immune microenvironment and immune status in the brain through the production of metabolites, direct immunomodulatory effects, and neuroendocrine signaling pathways, establishing it as an emerging therapeutic target in glioma management.

Gut microbiota-derived metabolites, particularly short-chain fatty acids (SCFAs) such as acetate, propionate, and butyrate, exert profound regulatory effects on central immune responses. These metabolites contribute to the maintenance of blood-brain barrier (BBB) integrity, thereby preventing the translocation of deleterious substances and pro-inflammatory mediators into brain parenchyma. In addition, SCFAs influence the polarization of tumor-associated macrophages (TAMs), favoring the accumulation of anti-tumorigenic M1-like macrophages, which enhances tumor immune surveillance and suppresses glioma growth (97, 98). Furthermore, butyrate derived from microbial metabolism functions as a potent histone deacetylase (HDAC) inhibitor, thereby augmenting the cytotoxic activity of CD8+ T cells and potentiating the therapeutic efficacy of immune checkpoint inhibitors (e.g., anti-PD-1 antibodies). These observations highlight the pivotal role of gut-derived metabolites in reshaping the glioma immune microenvironment (99).

Mechanistically, gut microbiota-derived metabolites may influence the glioma immune microenvironment through both direct and indirect routes. On the one hand, certain low-molecular-weight metabolites—particularly SCFAs such as acetate and propionate—can enter the systemic circulation and cross the BBB via passive diffusion or monocarboxylate transporter-mediated transport, thereby directly affecting resident CNS cells including microglia and astrocytes (76, 98, 100). Through these mechanisms, microbial metabolites can modulate histone acetylation, inflammatory signaling, and innate immune activation within the brain. On the other hand, many gut-derived metabolites appear to act predominantly through indirect immune pathways. For example, SCFAs, bile acid derivatives, and tryptophan metabolites regulate dendritic cell maturation, T-cell differentiation, and myeloid cell function in peripheral lymphoid tissues, thereby shaping systemic immune tone and influencing the phenotype of immune cells that subsequently infiltrate glioma lesions (76–78, 101, 102). In addition, glioma-associated disruption of BBB integrity may further facilitate the entry of circulating metabolites, cytokines, and immune mediators into the tumor region, amplifying gut-brain immune crosstalk (103–105). Therefore, the influence of gut microbiota on glioma should be viewed as a multilayered process involving direct metabolic signaling within the CNS, peripheral immune education, and BBB-dependent modulation of molecular trafficking.

Conversely, gut microbiota dysbiosis is increasingly recognized as a critical driver of immunosuppressive microenvironment formation in glioma. Antibiotic-induced dysbiosis reduces microbial diversity and SCFA production, leading to an elevated proportion of M2-like immunosuppressive macrophages within the tumor microenvironment (TME) and accelerating glioma progression (98, 106). Dysbiosis also induces systemic chronic inflammation, disrupts immune homeostasis, impairs the function of natural killer (NK) cells and T cells, and further reinforces tumor immune evasion (103). Moreover, microbiota perturbations alter the metabolism of neurotransmitters and essential amino acids (e.g., glutamate and tryptophan), which indirectly sculpt the tumor immune microenvironment by modulating immune cell proliferation and differentiation (107).

An expanding body of evidence from Mendelian randomization analyses and systematic reviews has established causal links between specific gut microbial taxa and glioma risk as well as immune phenotypes. For instance, genera such as Bacteroides and Faecalibacterium regulate the proportions of immune cells in peripheral blood and within the tumor, thereby influencing CD8+ T cell and regulatory T cell (Treg) function, modulating glioma immune responses, and impacting patient prognosis (108). These findings provide a strong rationale for exploiting the gut microbiota as early diagnostic biomarkers and adjunctive targets to augment immunotherapy in glioma.

In summary, the gut-brain axis modulates the glioma immune microenvironment via gut microbiota-derived metabolites and signaling pathways, conferring both opportunities for anti-tumor immune enhancement and risks of immunosuppression in the context of dysbiosis. Future research should prioritize delineating the precise molecular mechanisms underlying the immunomodulatory effects of specific microbial taxa and metabolites in glioma, while developing targeted microbiota interventions—such as probiotics, dietary modulation, and metabolite supplementation—to optimize immunotherapy responses and improve patient outcomes (104, 109, 110).

### 6.2 Gut microbiota metabolism in regulating immunotherapy potential

The gut microbiota serves as a critical regulator of host immunity, with its composition and derived metabolites exerting profound effects on the efficacy of tumor immunotherapy. Accumulating evidence demonstrates that modulating the gut microbiota structure and its metabolic activity can enhance responses to immune checkpoint inhibitors (ICIs) and other immunotherapies, while offering novel strategies to overcome inter-individual variability and acquired resistance.

For example, in hepatocellular carcinoma (HCC) patients, the gut microbiota influences immune responses via the gut-liver axis, with key metabolites—including bile acids, short-chain fatty acids (SCFAs), and bacterial toxins—participating in tumor progression and immune modulation. Microbiota-targeted interventions have been shown to improve ICI response rates and reduce treatment resistance (111). Moreover, microbial diversity and the abundance of specific taxa are closely correlated with immunotherapy outcomes. A variety of interventions, including probiotics, fecal microbiota transplantation, antibiotics, and natural compounds, have emerged as cutting-edge approaches to enhance immunotherapy efficacy (112, 113).

Notably, natural polysaccharides such as fucoidan have been found to restructure the gut microbiota, thereby potentiating the anti-tumor activity of anti-PD-1 monoclonal antibodies. The primary mechanisms involve enriching beneficial bacteria (e.g., Bifidobacterium and Lactobacillus), elevating SCFA levels (acetate and butyrate), enhancing effector T cell function, suppressing regulatory T cell (Treg) generation, and ultimately improving the immune microenvironment (114). Dietary factors, as a major modifiable regulator of the gut microbiota, have also been validated to promote the growth of beneficial microbes and their metabolic activity, thereby augmenting tumor immunotherapy efficacy (115). Traditional Chinese medicine formulations, such as Shenling Baizhu Powder, similarly enhance anti-PD-1 effects and mitigate immune-related adverse events by modulating the gut microbiota and its metabolites, promoting fatty acid metabolism, and demonstrating the potential for integrating traditional medicine with modern immunotherapy (116). Gut microbiota-derived metabolites—including SCFAs, bile acids, and tryptophan catabolites—regulate immune cell function through multiple immune-related signaling pathways, positioning them as novel targets in immunometabolic therapy. For instance, microbiota-mediated tryptophan metabolism activates the aryl hydrocarbon receptor (AhR), modulating immune tolerance and inflammatory responses while promoting anti-tumor immunity (101, 102).

In conclusion, the gut microbiota and its metabolites significantly shape tumor immunotherapy outcomes by regulating host immunity. These elements hold considerable promise for development as key targets and adjunctive strategies in precision immunometabolic therapy (117, 118).

## 7 Emerging technologies in the study of the glioma metabolic-immune axis

### 7.1 Single-cell and spatial transcriptomics reveal metabolic-immune heterogeneity

The advent of single-cell RNA sequencing (scRNA-seq) has dramatically advanced the detailed dissection of intratumoral cellular heterogeneity, particularly with respect to metabolic states and immune phenotypes. In glioma and other tumor types, scRNA-seq enables the identification of differential metabolic activities across cellular subpopulations, such as distinct activation patterns of energy metabolism pathways in tumor cells, immune cells, and stromal cells (8, 119). Studies have demonstrated the presence of metabolically diverse cell populations in glioma, including tumor cell clusters characterized by high glycolytic activity and immune cell subsets predominantly reliant on fatty acid oxidation, collectively forming a complex metabolic network (8, 119). Concurrently, the metabolic status of immune cells directly governs their activation and functional output; for instance, tumor-associated macrophages (TAMs) in the tumor core exhibit enhanced oxidative phosphorylation and ATP synthesis, thereby facilitating tumor progression (120). Furthermore, scRNA-seq has uncovered metabolic reprogramming within the tumor microenvironment (TME), exemplified by heightened lipid metabolism and immunosuppressive functions in M2-polarized macrophages (121, 122). This metabolic heterogeneity offers novel insights into the mechanisms underlying tumor immune evasion.

Spatial transcriptomics complements the limitations of scRNA-seq in spatial resolution, enabling researchers to observe, within the native tissue environment, the spatial associations between metabolically active regions and the distribution of immune cells. For example, in glioma and other solid tumors, spatial transcriptomics has revealed co-localization of high metabolic activity in the tumor core with infiltration of immunosuppressive cells, suggesting that metabolic reprogramming may drive local immunosuppression through microenvironmental alterations (122, 123). Integration of spatial metabolomics with spatial transcriptomics has further delineated intricate metabolic exchange networks between tumor and immune cells, emphasizing that metabolically active tumor regions are frequently associated with immune exclusion or restricted immune cell functionality (124, 125). Additionally, spatial transcriptomic analyses have elucidated key spatial distribution patterns of stromal cells, such as cancer-associated fibroblasts (CAFs), in tumor metabolism and immune regulation, thereby deepening understanding of immunosuppressive mechanisms within the TME (125, 126).

In summary, single-cell RNA sequencing focuses on inter-cellular heterogeneity in metabolic and immune phenotypes, whereas spatial transcriptomics elucidates the spatial relationships between these metabolic features and immune cell distribution. Their combined application provides an unprecedented high-resolution perspective on the molecular mechanisms of the glioma metabolic-immune axis, accelerating the development of metabolically targeted immunotherapeutic strategies, enabling precise spatial localization, and advancing individualized clinical treatment approaches (8, 122, 123).

### 7.2 Stable isotope tracing for dissecting metabolic pathways

Stable isotope tracing has emerged as a powerful and versatile tool for dissecting tumor metabolic pathways and their interactions with immunity, demonstrating substantial potential in glioma and other cancer metabolism research in recent years. This technique involves the introduction of metabolically labeled substrates containing stable isotopes (e.g., ¹³C-, ¹^5^N-, or ²H-labeled glucose or glutamine), followed by tracking of label incorporation, flux, and distribution through intracellular pathways using high-sensitivity analytical platforms such as mass spectrometry or nuclear magnetic resonance. This approach reveals dynamic features of metabolic flux. It is particularly well-suited for near-physiological culture conditions, such as human plasma-like medium (HPLM), which more faithfully recapitulates the metabolic states of tumor cells and their microenvironment compared to conventional media, thereby overcoming artifacts induced by non-physiological culture conditions (24, 25).

In glioma research, stable isotope tracing not only elucidates intrinsic metabolic activity in tumor cells but also dissects metabolic features and inter-cellular regulation within the tumor microenvironment (TME). For example, tracing with ¹^5^N^2^-glutamine in glioma tissue slices revealed tumor-specific purine synthesis activity in neoplastic cells and distinctive GDP-mannose metabolism in tumor-associated astrocytes, highlighting complex metabolic heterogeneity and intercellular metabolic division of labor (25). Furthermore, integration of stable isotope tracing with spatial metabolic imaging enables dynamic, spatially resolved observation of metabolite distribution and flux within intact tissue, further delineating metabolic heterogeneity in glioma, kidney, and breast tumors—including the spatial dynamics of key pathways such as glycolysis, the tricarboxylic acid (TCA) cycle, and the pentose phosphate pathway (127).

Stable isotope tracing also plays a pivotal role in dissecting crosstalk between tumor metabolism and immune metabolism. By combining multiple isotopically labeled substrates in near-physiological culture systems, this method systematically elucidates how tumor metabolic reprogramming influences immune cell energy supply and functional status, as well as how immune cell metabolism reciprocally modulates tumor metabolism, thereby advancing a holistic understanding of the tumor-immune metabolic axis (25). This approach provides a robust experimental foundation for in-depth exploration of tumor immune evasion mechanisms and identification of metabolic vulnerabilities.

Advances in downstream data processing and analysis, including software platforms such as Escher-Trace and Khipu-web, have greatly facilitated the integration and visualization of stable isotope tracing data. These tools enable mapping of complex isotopologue labeling patterns onto established metabolic networks, facilitating the identification of key metabolic nodes and regulatory mechanisms (128, 129). Additionally, algorithms for correcting natural isotopologue abundances, such as PolyMID-Correct, have enhanced data accuracy and reliability (130).

Despite its considerable strengths, stable isotope tracing is subject to several important technical limitations that must be acknowledged for rigorous interpretation. First, isotopic steady-state assumptions may not always be met in complex biological systems, particularly in heterogeneous tumor tissues where metabolic rates vary substantially across cell subpopulations; non-steady-state conditions can confound flux calculations derived from isotopologue distributions (131). Second, natural isotopologue abundance correction, while addressable by algorithms such as PolyMID-Correct, remains a potential source of error, particularly for low-abundance metabolites where signal-to-noise ratios are limiting (130). Third, the use of supraphysiological substrate concentrations (e.g., uniformly labeled ¹³C-glucose at concentrations deviating from plasma levels) can artificially alter metabolic routing and produce isotopologue patterns that do not accurately reflect *in vivo* fluxes; this concern is partially mitigated by the adoption of human plasma-like medium (HPLM), though HPLM itself may not perfectly recapitulate the dynamic and spatially heterogeneous metabolic environment within intact tumors (24, 25). Fourth, stable isotope tracing in intact tissues or *in vivo* systems faces challenges in distinguishing cell type-specific contributions to observed isotopologue enrichments without coupling to single-cell or spatially resolved analytical platforms, which themselves remain technically demanding and not yet widely accessible (131). Fifth, the approach provides information primarily on relative metabolic fluxes rather than absolute metabolite concentrations, requiring complementary quantitative metabolomics for comprehensive metabolic characterization. These limitations underscore the importance of integrating stable isotope tracing with orthogonal methodologies—including spatial metabolomics, single-cell multi-omics, and computational metabolic modeling—to obtain a comprehensive and accurate picture of tumor metabolic dynamics.

In summary, the application of stable isotope tracing in conjunction with human plasma-like medium provides a formidable methodological framework for investigating glioma metabolism and its interactions with the immune system. This technique precisely captures metabolic features across diverse cell types within the tumor and its microenvironment, reveals spatial and temporal dynamics of metabolic pathways, and establishes a foundation for understanding tumor metabolic heterogeneity and the regulatory mechanisms of the metabolic-immune axis. It holds considerable translational value and clinical potential. As technological and analytical capabilities continue to advance, stable isotope tracing is poised to play an increasingly central role in tumor metabolism research and precision oncology (24, 25, 131).

## 8 Conclusion

Glioma, as one of the most challenging malignant tumors of the central nervous system, is profoundly shaped by a complex metabolism-immunity axis that plays a central role in tumor initiation, progression, and clinical treatment. Metabolic reprogramming not only provides tumor cells with the energy and biosynthetic resources required for sustained proliferation but also regulates the immunosuppressive microenvironment through multiple mechanisms, thereby facilitating immune evasion. From a comprehensive and multidimensional perspective, it is essential to evaluate developments in this field by balancing mechanistic insights from different research groups with clinical applicability, ultimately promoting innovation and optimization in glioma therapy.

First, integrative multi-omics classification and metabolism-based risk models have emerged as important tools in precision medicine. Studies employing genomics, transcriptomics, metabolomics, and other multi-layered datasets have constructed molecular subtypes and metabolism-related prognostic models for patients with glioma. These models not only improve the accuracy of prognostic prediction but also provide a scientific basis for the design of individualized therapeutic strategies. Nevertheless, substantial heterogeneity exists across different cohorts and technical platforms. Therefore, achieving data standardization and robust validation through large-scale, multi-center collaborative studies represents a critical direction for future research.

Second, the role of metabolic products in regulating immune evasion has become increasingly evident. Lactate, tryptophan metabolites, and fatty acid derivatives not only influence the functional states of immune cells but also reshape the immunosuppressive microenvironment through crosstalk between metabolic and signaling pathways. Several studies have proposed immunometabolic combination strategies aimed at reversing immune suppression by targeting metabolic pathways and thereby enhancing the efficacy of immunotherapy. Although promising effects have been observed in preclinical studies, the safety, tolerability, and long-term efficacy of immunometabolic interventions require further validation in clinical trials. Establishing a closer translational bridge between experimental models and clinical research will therefore be critical to ensure that theoretical advances can be effectively implemented in clinical practice.

The emerging association between gut microbiota-derived metabolism and immune regulation in glioma provides a new perspective for therapeutic exploration. The gut microecosystem modulates host immunity through its metabolic products and thereby participates in shaping the tumor immune microenvironment. Although this field remains in its early stages, integrating microbiota-based regulation with the metabolism-immunity axis highlights the importance of multisystem interactions and supports the exploration of combinatorial regulatory strategies to improve therapeutic outcomes.

Technological advances have provided powerful tools for a deeper understanding of the glioma metabolism-immunity axis. Single-cell sequencing has revealed the cellular heterogeneity of tumors and their microenvironment, spatial omics technologies have elucidated the spatial distribution of metabolic and immune events, and stable isotope tracing has enabled characterization of metabolic fluxes and dynamic changes. These emerging approaches not only expand our understanding of glioma biology but also provide a data-driven foundation for the development of precision therapeutic strategies. In the future, artificial intelligence-based integration of multimodal data is expected to further enhance our understanding of complex metabolic-immune networks and accelerate the advancement of individualized precision medicine.

In summary, research on the glioma metabolism-immunity axis is rapidly evolving and demonstrates substantial potential in both mechanistic discovery and clinical translation. Interdisciplinary collaboration and multisystem integration should be emphasized to advance combined strategies targeting metabolism and immunity, alongside the development of large-scale, standardized clinical trial frameworks. Meanwhile, future studies should focus on the crosstalk between metabolic pathways and immune regulation, elucidate the specific roles of metabolic products in immune escape, and explore more effective combinatorial therapeutic strategies, ultimately improving clinical outcomes for patients with glioma. At a critical juncture in the transformation of global oncology, investigation of the glioma metabolism-immunity axis is poised to become a key breakthrough in precision cancer immunotherapy and to offer new hope for patient survival.
